# All‐in‐One Protocol: A Comprehensive Sedation Approach for Multi‐Procedural Aesthetic Dermatology

**DOI:** 10.1111/jocd.70400

**Published:** 2025-08-16

**Authors:** Daniel C. Dziabas, Matheus M. de S. Kasai, Marina Ajeje Lobo, Marcus Vinicius Esclavacini, Gisele Chicone

**Affiliations:** ^1^ Dziabas Academy & Research Institute São Paulo Brazil; ^2^ Azoto Anestesia São Paulo Brazil

**Keywords:** aesthetic procedures, All‐in‐One protocol, anesthesia in dermatology, pain management, sedation

## Abstract

**Background:**

Common aesthetic procedures, including High‐Intensity Focused Ultrasound (HIFU), microneedling with radiofrequency, and laser treatments, are effective but often associated with significant pain. Traditional anesthesia techniques, such as topical creams and conscious sedation, provide limited pain relief, affecting patient comfort and treatment intensity. The All‐in‐One protocol, leveraging sedation, overcomes these limitations by enabling practitioners to deliver pain‐free treatments and with higher energy settings, thus improving clinical outcomes.

**Objectives:**

The aim was to develop a protocol for dermatological aesthetic procedures using sedation in a safe way, guaranteeing the effectiveness of treatments and reducing the total number of sessions, minimizing downtime.

**Methods:**

This study was conducted in a private dermatology clinic in São Paulo to develop a protocol for safely using sedation, guaranteeing the effectiveness of treatments and reducing the total number of sessions, minimizing downtime. The protocol was drawn up after analyzing the literature to implement sedation.

**Results:**

The All‐in‐One protocol combined procedures such as HIFU, lasers, and injectables with higher energy settings possible under sedation due to the elimination of pain. Patients are continuously monitored throughout, following regulatory guidelines for office‐based sedation.

**Conclusion:**

The All‐in‐One protocol represents a safe and innovative solution for performing multiple high‐energy aesthetic procedures in a single session under sedation. It enhances patient comfort and improves outcomes, allowing more effective treatments with minimal downtime. With appropriate safety measures, this method offers a superior alternative to conventional anesthesia techniques, providing a streamlined, patient‐centric approach to comprehensive aesthetic care.

## 1 Introduction

Modern aesthetic dermatology has advanced significantly with the development of energy‐based technologies and injectable treatments. While many aesthetic procedures can be performed nonsurgically, they are not without challenges and can be associated with significant discomfort, which can limit their application, patient satisfaction, and overall well‐being [1, 2].

The realm of dermatological treatments has witnessed a surge of innovative technologies, each promising remarkable results in addressing various skin concerns. However, the pursuit of enhanced efficacy has often come at the expense of patient comfort, with certain procedures such as high‐intensity focused ultrasound (HIFU), radiofrequency microneedling, and fractionated lasers being associated with significant discomfort and the need for anesthesia [3, 4]. When evaluating the use of HIFU for face and neck rejuvenation, the authors found in a meta‐analysis that the average pain score was 4.2, assessed by a 0–10 Likert scale (95% CI 4.27–5.19) and showed moderate overall satisfaction 2.28 (Likert scale 0–5; 95% CI 0.77–3.80) at 6 months [5]. In the other study, the radiofrequency microneedling the mean pain intensity was 5.6/10 on the Numeric Rating Scale [6]. For acne vulgaris treatment with the fractional CO_2_ laser, the experience of mean pain was 8 by 10‐point visual analog scale [7].

The management of patient discomfort during medical procedures is a critical concern in healthcare. Oftentimes, the pain and discomfort lead to a reduction in equipment parameters, such as power and energy levels, to mitigate their suffering [8, 9]. This reduction in treatment parameters, however, can compromise the effectiveness of the procedure and the overall success of the treatment. Patients may even refuse to undergo certain steps of the treatment or decline the treatment altogether due to the memory of past pain, further jeopardizing the desired outcomes [10].

Currently, aesthetic procedures are expected to be performed nearly pain‐free. Analgesia and anesthesia prevent the patient from the physical sensation of pain. Often, it is overlooked that individuals perceive pain to different extents, and these individual differences are of importance when it comes to the interpretation of pain experienced during medical interventions [2, 9, 11].

Conventional methods of pain control, such as topical anesthetics, offer only superficial pain relief and are associated with risks of systemic absorption and toxicity, especially when applied over large areas. Anesthetic topics, such as lidocaine and benzocaine, should be applied beforehand and reduce the sensation of pain [12, 13]. Efficacy can vary and depends on the individual's perception. There may also be local reactions [13], such as whitening and erythema, or systemic intoxication, such as dizziness, drowsiness, tinnitus, and perioral paresthesia. More severe symptoms include disorientation, delirium, convulsions, coma (at concentrations of 10–20 mg/L) and cardiorespiratory arrest (at concentrations above 20 mg/L) for lidocaine cream [14]. This can occur mainly when the cream is applied to large areas, which is very common during tattooing, or if the skin is damaged or compromised.

Local anesthetics, such as lidocaine, are commonly used to numb specific areas of the body, particularly in more sensitive regions or for larger medical procedures. However, this approach is limited by the requirement for medical expertise to administer the injections, as well as the associated pain and risk of systemic intoxication [15, 16].

A safe alternative is sedation, which is a less invasive anesthetic technique than general anesthesia, as it uses lower doses of medication, maintaining a less profound level of consciousness (Bispectral Index [BIS] between 60 and 80) and allowing spontaneous breathing without the need for mechanical ventilation, as well as faster awakening in the postsurgical period. For nonpainful procedures, sedation aims to reduce anxiety and control movement. Conscious sedation, on the other hand, provides limited pain relief and may leave the patient partially aware and uncomfortable during the procedure [17]. These limitations highlight the need for a more effective approach to anesthesia in aesthetic dermatology, exploring alternative methods for achieving more comfort for patients [18, 19].

With the patient sedated and pain‐free, there is increased relaxation and decreased anxiety, which allows the use of dermatological devices with greater depth and intensity. This leads to the best possible aesthetic result within the available technologies, a key advantage of conscious sedation [20]. Studies suggest that rapid recovery and minimized side effects are significant advantages of conscious sedation [21, 22].

From a scientific point of view, patients are always looking for the best option when seeking an aesthetic procedure; this information could be a key factor since the procedure is beneficial in relation to pain management and the satisfaction of the patients involved [23]. The study aimed to develop a protocol for the management of pain and functioning during the application of aesthetic procedures. The All‐in‐One protocol addresses these challenges by integrating multiple procedures in a single session under sedation. This approach allows dermatologists to perform treatments with greater energy settings and precision, while ensuring a pain‐free experience for the patient.

## 2 Materials and Methods

The All‐in‐One protocol was drawn up after analyzing the literature to implement sedation for aesthetic procedures in a private dermatology clinic in São Paulo, Brazil.

## 3 Results

The All‐in‐One protocol provides sedation which is administered by a licensed anesthesiologist in a fully equipped dermatology office. This approach is often referred to as “twilight anesthesia” because the patient remains unaware of the procedure but is not completely unconscious. Drugs such as propofol, midazolam, or dexmedetomidine are commonly used in this setting due to their rapid onset of action and short half‐life, allowing for quick recovery times [19].

### 3.1 Procedure Selection

The All‐in‐One technique is well‐suited for energy‐based technologies, which can cause pain, and injectable treatments that benefit from higher energy settings and precision. Procedures such as HIFU, fractional laser resurfacing as CO_2_, Erbium, Superb (Synchronous Ultrasound Parallel Beam SUPERB), radiofrequency microneedling, subcutaneous lasers, and injectable fillers, biostimulators of collagen, botulinum toxin, or PDO/PCL/PLLA threads are ideal candidates for this approach. By eliminating pain, the practitioner can increase the intensity of energy‐based treatments, resulting in improved efficacy with fewer sessions. In addition, multiple areas of the face and body can be treated in a single session, providing a more comprehensive aesthetic result in a shorter time frame.

### 3.2 Patient Assessment and Selection

A tailored preanesthetic assessment is conducted several days prior to the procedure. We contact the patient beforehand for an interview, asking about their surgical/anesthetic history, allergies, comorbidities, medications in continuous use, instructions on fasting beforehand, and other essential information to ensure the procedure's safety. A detailed anamnesis evaluation is crucial for identifying possible complications and adequately preparing the anesthetic plan [24].

Before the procedure begins, the patient must be carefully assessed by the anesthesiologist in charge. This assessment should include a detailed anamnesis and physical examination, seeking to identify risk factors such as the American Society of Anesthesiologists (ASA) classification, which is used to assess the patient's physical condition before surgical and anesthetic procedures [25]. In addition, investigate facial dysmorphisms (Mallampati III and IV), large tonsils, heart or respiratory diseases, mediastinal mass, dental appliances or loose teeth, chromosomal abnormalities, obesity, sleep apnea, drug allergies, previous problems with sedation or anesthesia, presence of a difficult airway, and respiratory infections. Identifying these factors is essential to minimize risks and ensure patient safety during the procedure [26].

For safety reasons, procedures should not exceed 5 h. The anesthesiologist remains in the clinic for the entire time.

Sedation can be indicated for different types of patients, depending on their medical condition and the procedure. However, there are criteria for determining who can be safely sedated [27]. Patients who can generally make use of sedation include:

- Healthy adults without significant medical problems or comorbidities treated and/or under treatment, especially those classified as ASA I and ASA II (Table 1).
- Patients with anxiety or phobia.
- Patients with special needs: people with physical, cognitive, or behavioral conditions that make it difficult to cooperate during procedures.
- Healthy elderly or elderly with controlled comorbidities.

**TABLE 1:** American Society of Anesthesiologists physical status classification system.

ASA physical status classification	Definition
I	A normal healthy patient
II	A patient with mild systemic disease
III	A patient with severe systemic disease
IV	A patient with severe systemic disease that is a constant threat to life
V	A moribund patient who is not expected to survive without the operation
VI	A declared brain‐dead patient whose organs are being removed for donor purposes

*Note:* Adapted and summarized version of the ASA classification (American Society of Anaesthesiologists physical status classification system) [25].

The main contraindications [27] for the use of sedation include:

- Severe heart disease as heart failure, uncontrolled arrhythmias, or severe coronary disease.
- Severe respiratory diseases include severe asthma, chronic obstructive pulmonary disease (COPD), untreated sleep apnea, or respiratory failure.
- Allergy or hypersensitivity to sedatives.
- Pregnancy.
- Patients with severe hepatic or renal dysfunction.
- History of adverse reactions to anesthesia or sedation.
- Uncontrolled serious psychiatric disorders as schizophrenia or bipolar disorder in crisis can make cooperation difficult.
- Frail elderly or those with multiple comorbidities.

### 3.3 Sedation Technique

The sedation plan is meticulously individualized, factoring in the patient's medical history, physical status, and procedural requirements.

#### 3.3.1 Monitoring

Prior to sedation, patients are connected to advanced monitors to continuously track vital parameters such as heart rate, blood pressure, and oxygen saturation. During the procedure, the depth of sedation and the patient's vital signs are monitored continuously. Medication dosages are adjusted dynamically to maintain optimal comfort and ensure patient safety throughout the procedure.

#### 3.3.2 Medication Administration

- Midazolam: This is initially administered to reduce anxiety and induce mild to moderate sedation. The initial dose for a 70 kg patient usually ranges from 1 to 2 mg intravenously and can be adjusted as necessary [24].
- Fentanil: It is used to provide analgesia. The starting dose for a 70 kg patient is 0.5–1 μg/kg, administered slowly; that is, this dose would be equivalent to 35–70 μg for a 70 kg patient [18].
- Propofol: This is administered to induce deep sedation and maintain the sedative state. The initial dose is 0.5–1 mg/kg, 35–70 mg for a 70 kg patient. The maintenance dose is adjusted as necessary to maintain the desired level of sedation [27].

Symptomatic medications such as analgesics, antiemetics, anti‐inflammatories, and antibiotics are also used to avoid any foreseeable adverse effects.

### 3.4 Patient Recovery and Post‐Procedure Orientation

Following the procedure, patients are transferred to a recovery area for continuous monitoring by the anesthesiologist and the nursing team. During this phase, specific signs are continuously monitored. Patients are observed until full recovery of consciousness and stabilization of clinical parameters. Once this state is reached, a light meal is offered to aid metabolic recovery. Prior to discharge, patients are provided with comprehensive postprocedural care instructions and are released under the supervision of a designated companion, ensuring adherence to all recommendations.

### 3.5 Sedation Risks

Anesthetic sedation, like any medical procedure, involves risks that include:

- Allergic reactions.
- Respiratory complications, including depression, hypoventilation, or airway obstruction.
- Cardiovascular problems: sedation can lead to changes in blood pressure, arrhythmias, and in rare cases, heart attack.
- Neurological complications: (although rare) there may be a risk of temporary or permanent neurological damage.
- Pulmonary aspiration.
- Prolonged effects, such as confusion, dizziness, or extended sedation periods.

### 3.6 Patient Comfort and Satisfaction

The protocol was applied in 42 patients. The patient satisfaction was assessed using the Global Aesthetic Improvement Scale (GAIS) and a customized pain and comfort questionnaire. GAIS is a universal scale that has been used in several studies to monitor the level of improvement in facial aesthetics and treatment results [28, 29, 30]. More than 90% of patients rated their experience as ‘excellent’ citing minimal discomfort and high satisfaction with the outcomes. The observed adverse events were minimal and aligned with the expected outcomes for the procedures performed, comprising transient localized edema, ecchymosis in areas subjected to greater manipulation, and mild postprocedural discomfort, effectively managed with simple analgesics. Furthermore, one patient developed drowsiness following sedation and reported nausea without emesis. Patients underwent follow‐up evaluation 72 h postprocedure and at 30‐day intervals to monitor their progress and outcomes.

### 3.7 Safety Considerations and Equipment Requirements

To comply with regulatory standards for in‐office sedation, the treatment setting must be equipped with advanced monitoring equipment, emergency resuscitation tools, and personnel trained in sedation management. In the United States, this includes adherence to the guidelines set by the ASA and similar local guidelines in other regions, such as ANVISA in Brazil and the European Board of Anesthesiology in the EU. The office must have access to oxygen supply, defibrillators, and medications for the reversal of sedation if needed [26].

The use of sedation in aesthetic medicine must be carefully weighed against its potential risks and associated costs. While sedation can enhance patient comfort, reduce anxiety, and improve cooperation—particularly during more painful or prolonged procedures—it also presents inherent risks, including respiratory depression, nausea, vomiting, and, in some cases, the need for enhanced monitoring. Therefore, its use should be approached with caution and tailored to the individual patient's clinical profile.

Sedation can lower the threshold of perceived discomfort, enabling the clinician to perform more extensive or aggressive interventions within a single session. However, this may also elevate the risk of adverse outcomes, such as bruise, edema, or infection complications. The decision to extend a procedure must always be carefully balanced among patient safety, anticipated aesthetic benefit, and the recovery process.

## 4 Discussion and Conclusion

In recent years, there has been an increase in the search for aesthetic procedures. In Brazil in 2022, more than two million surgeries were performed in this area, while in the USA, more than 5.8 million nonsurgical aesthetic procedures were performed [31]. Most of these minimally invasive procedures have been carried out in clinics, and the introduction of sedation in dermatology practices has opened the door to a more patient‐centric approach to aesthetic procedures.

By mitigating pain and enhancing tolerance for high‐intensity treatments, this approach enables practitioners to expand service offerings and achieve superior outcomes. Managing patient discomfort during aesthetic procedures is essential to provide better power and energy levels and promote these treatments' effectiveness and success without potentializing their suffering [19].

The All‐in‐One protocol is particularly beneficial for patients seeking combined treatments in a single session in a limited time frame. Although there is a great demand for it, published studies demonstrating the use of sedation in nonsurgical aesthetic procedures are limited. Much of the literature refers to the use of sedation and local anesthesia in surgical procedures, even if performed outside the hospital. In a 6‐year retrospective cohort of surgical procedures carried out in a clinic, most patients were women who underwent 283 facial aesthetic surgical procedures. There were 195 patients in ASA class I and four in ASA class II. During the study period, there were no significant complications and no sedation issues [17]. In 2002, Massimo [32] already reported on the combination of procedures and the use of sedation to improve the results of plastic surgeries. In patients treated with All‐in‐One protocol, we have observed improvements in results ranging from 25% to 50%, along with a reduction in the number of sessions of nearly 50%, depending on the associated procedures.

The growing number, variety, and complexity of cases increase patient safety concerns. At the same time, the field of anesthesia has reached such an advanced stage that it is now possible to guarantee a high level of patient comfort and safety in all procedures. The skills of anesthesiologists and today's resources make it possible to plan appropriately for each type of intervention. Studies show that the choice of sedation must consider several factors, including the duration of the procedure, the patient's general health, and the level of invasiveness [18, 24].

Therefore, the safety profile of sedation in the context of aesthetic dermatology is strong, with minimal risks when proper protocols are followed. Continuous monitoring of the patient during the procedure ensures that any potential complications can be addressed immediately. Additionally, the rapid recovery time associated with sedation allows patients to return to their daily activities sooner, further enhancing the appeal of this approach [22, 32]. Moreover, when the patient feels no pain, there is no trauma associated with the procedure either, and this experience becomes comfortable, allowing them to return to the health service for treatment again [33, 34]. The ability to tolerate higher energy settings during procedures led to more pronounced and long‐lasting aesthetic improvements.

In many areas, there is a debate about pain through the evaluation of adverse impacts faced by patients undergoing such procedures and what they desire for their aesthetic experience. The developed protocol saves relevant information for the clinical practice of health professionals, contributes to the care of patients undergoing aesthetic procedures, and establishes boundaries that should not be crossed in medical practices, enhancing empowerment and access to safety in evaluating such experiences. Therefore, the patient can contemplate aesthetic procedures safely and with excellent results.

Despite the many benefits, the biggest challenge was structuring safety protocols and training that met regulatory requirements and patients' expectations, who traditionally associated dermatology clinics with quick and minimally uncomfortable procedures. Creating specific internal flows for preanesthetic assessment, monitoring, and postanesthetic support was fundamental to the success of the implementation.

The introduction of the anesthesia service required adjustments to the physical and operational structure of the clinic but did not involve creating our anesthesiology team. We opted to hire an outsourced anesthesia service, which allowed for greater flexibility, cost control, and financial viability for the implementation. Although there has been an increase in the individual cost of each procedure, this amount is passed on transparently to the patient, balancing the investment with the safety and comfort provided.

In addition, it was necessary to align patients' perceptions with the reality that multiple procedures involve greater complexity than isolated treatments when carried out in the same session to optimize results. It was necessary to reinforce the communication that anesthesia was aimed at increasing safety, comfort, and the efficiency of results, demystifying the idea that it would be excessive or unnecessary.

So All‐in‐One protocol provides an effective framework for integrating multiple aesthetic procedures into a single, sedation‐supported session. This technique improves patient comfort, allows for the use of higher energy settings, and leads to superior clinical outcomes.

## Author Contributions

M.M.S.K. and G.C. performed the research, designed the research study, analyzed the data and wrote the paper. D.C.D. designed the research study and reviewed the paper. M.A.L. and M.V.E. contributed with review and tools to the paper.

## Conflicts of Interest

Daniel C. Dziabas is a speaker for Galderma, Merz Aesthetics, IBSA Derma, Entera, and LMG Lasers and has participated in lectures and medical events for these brands. Matheus M. de S. Kasai is a scientific consultant for Ilikia Brasil and has participated in lectures, consulting, and medical events. Marina Ajeje Lobo declares no conflicts of interest. Marcus Vinicius Esclavacini declares no conflicts of interest. Gisele Chicone declares no conflicts of interest.

## Data Availability

The data that support the findings of this study are available from the corresponding author upon reasonable request.
